# The 10-D assessment and evidence-based medicine tool for authors and peer reviewers in clinical pharmacology 

**DOI:** 10.5414/CP203073

**Published:** 2017-07-03

**Authors:** Barry G. Woodcock, Sebastian Harder

**Affiliations:** 1 Dustri Medical Science Publications, Dustri-Verlag Dr. Karl Feistle GmbH & Co. KG, Deisenhofen-Munich, Germany, and Dustri-Verlag, Inc., Rockledge, FL, USA, and; 2 Institute of Clinical Pharmacology, pharmazentrum frankfurt, University Hospital, Johann Wolfgang Goethe-Universität, Frankfurt am Main, Germany

**Keywords:** peer review, clinical pharmacology, 10-D assessment, evidence-based medicine, Good Publication Practices (GPP), registration of observational studies

## Abstract

Background: Peer reviewers and authors of clinical pharmacology manuscripts need to meet the standards for Evidence-Based Medicine (EBM) and Good Publication Practices (GPP), and editors of clinical pharmacology journals have to maintain an overview of the peer review process. Methods and results: The peer review process can be monitored and facilitated using the **10-D assessment,** which comprises peer review criteria to determine if: 1. **d**esign of the study, 2. **d**iagnoses employed, 3. **d**rug molecules involved, 4. **d**osages applied, 5. **d**ata collected, 6. **d**iscussion of the findings, 7. **d**eductions made, 8. **d**ocumentation, 9. **d**eclarations, and 10. **d**HS (drug hypersensitivity syndrome) risk assessment is in accord with the objectives of the study and meet the requirements of EBM and GPP. Conclusions: The **10-D assessment** tool, although easy to apply, requires a high level of clinical pharmacology expertise, especially in the fields of drug disposition, pharmacokinetics, and drug action. Its application will facilitate the peer review of clinical research and clinical trial reports and thus promote safety in drug development and pharmacotherapy and meet the needs of Good Publication Practices.

The herein described **10-D assessment** tool for peer reviewers, authors, and publishers of clinical pharmacology manuscripts is a further development of the 7-D assessment [1] and 8-D assessment reported previously [2]. 

The need for such a tool was highlighted recently in an article with the rather ominous title *“Organized crime against the academic peer review system”* [3]. The problem was that some editors had lost their overview in managing manuscripts submitted to their journals to the extent that some authors had been able to “peer review” their own manuscripts [4]. A retraction was subsequently published after the article had appeared in print. 

In the case of clinical pharmacology, peer review criteria should be suitable for manuscripts covering a wide variety of topics, research protocols, and manuscript formats. As well as being clearly defined, comprehensive, and dealing with the aspects important in clinical pharmacology, these criteria must be simple to apply. The publication of clinical findings is a driving force in pharmacotherapy, and therefore the peer review process is a determinant for safety in drug development and pharmacotherapy. The peer review process must therefore meet the requirements of Evidence-Based Medicine (EBM) [5] and Good Clinical Practices (GPP) [1]. 

The **10-D assessment** evaluates whether the following criteria:

1. **d**esign of the study,
2. **d**iagnoses employed,
3. **d**rug molecules involved,
4. **d**osages applied,
5. **d**ata collected,
6. **d**iscussion,
7. **d**eductions made
8. **d**ocumentation support
9. **d**eclarations
10. **d**HS risk assessment

are in accord with the objectives of the study, meet the requirements of EBM and Good Publication Practices (GPP) [1] as well as the criteria for registration and publication of observational studies in humans (see Appendix) where: 

*Right design* means that the study design and protocol are appropriate for answering the question(s) being asked.

*Right diagnosis* is relevant for investigations both in patients and healthy subjects where subject and patient description and patient selection need to be detailed, accurate, and appropriate for the aims of the study.

*Right drug molecule* begs the questions, “Is the active agent a known molecular species?” and “Can the drug entity have a mode of action compatible with the observed pharmacological effects? Does a pharmacological effect observed in vitro have a counterpart in vivo? Do confounding factors, such as the presence of drug enantiomers, stereoisomers, or drug combinations, exist?” Herbal drugs and extracts do not generally fit in with the concepts of EBM. High first-pass effects make it likely that more than one active species is present in the tissues.

*Right dosage* concerns not only the size of the dose (i.e., is the dose or concentration clinically relevant?), but also the method of administration, bioavailability, and duration of treatment. These questions also apply to in vitro studies with tissues and cells.

*Right data* are those data required to meet the objectives of the study, which can establish or disprove efficacy, which have been obtained using state-of-the-art methods, and which have been evaluated using recognized data-analysis procedures. In the case of reviews of the literature, the retrieval methods used and quality of the studies reviewed need to be scrutinized.

*Right discussion* means that all limitations of the study are stated, new findings are highlighted, differences compared to other investigations are discussed satisfactorily, and due recognition is given to the work of earlier investigators in the field.

*Right deductions* means that conclusions are based on a correct and objective interpretation of the research findings and recommendations are made with due caution regarding patient safety and efficacy requirements in clinical pharmacotherapy.

*Right documentation* addresses primarily the quality of the evidence in the supportive literature and asks the questions: “Is the documentation up-to-date? Is it obtained from peer-reviewed sources, and is it comprehensive?” The citation of websites is very useful for providing information but must be viewed with caution when used to provide evidence. Information on websites is not peer reviewed and can be subject to change.

*Right declarations* means that, where appropriate, the research project or clinical trial has been conducted in accord with the Declaration of Helsinki, has been registered by the responsible authority, and registration details are stated in the abstract (see Appendix), an ethics commission (for human or animal studies), internal review board or external review board has been consulted, patient consent has been obtained correctly, conflicts of interest have been declared, and transparency exists regarding the contribution of individual authors.

*Right DHS risk assessment* addresses the question: “Has the possibility of a DHS (drug hypersensitivity syndrome, either IgE–mediated (immediate) or non-IgE-mediated (delayed), i.e., ADR Type B) that could occur during the study been addressed appropriately?” [6, 7, 8].

The risk can be graded 1 – 3 using: 

*1. No risk:* established drug in a host from a non-risk ethnic group. 

*2. Moderate risk: *a) innovative drug, or b) established drug from a risk category, or c) established drug used in a host from a risk ethnic group. 

*3. High risk: *a) innovative drug or b) drug from a risk category used in a host from a risk ethnic group and taking into account: 

*Drug Factors:* nature of the drug, degree of exposure (dose, duration, frequency), route of administration, cross-sensitization. Drugs frequently associated with DHS are aspirin (other analgesics-antipyretics), penicillins and cephalosporins, sulfonamides, and anticonvulsants.

and 

*Host factors: *age and sex, genetic factors (HLA type, acetylator status), concurrent medical illness (e.g., Epstein-Barr virus (EBV), human immunodeficiency virus (HIV), asthma), previous drug reaction, multiple allergy syndrome.

If the findings regarding any one of these assessment criteria are questionable, the compliance of the research with EBM and GPP principles is weakened, and the reviewers and editors will make recommendations accordingly. 

## Conclusion 

The **10-D assessment,** a tool to assist authors and peer reviewers of clinical pharmacology manuscripts to meet the requirements for GPP and EBM and to help editors of clinical pharmacology journals maintain an overview of the peer review process has been described. It comprises peer review criteria to determine if the: 1. **d**esign of the study, 2. **d**iagnoses employed, 3. **d**rug molecules involved, 4. **d**osages applied, 5. **d**ata collected, 6. **d**iscussion of the findings, 7. **d**eductions made, 8. **d**ocumentation supporting the work, 9. **d**eclarations concerning ethical and transparency questions, and 10. **d**HS risk assessment is in accord with the objectives of the study and meet the requirements of EBM and GPP. This tool, although easy to apply, requires a high level of clinical pharmacology expertise, especially in the fields of drug disposition, pharmacokinetics, and drug action. Its implementation will improve standards in publishing clinical pharmacological research, and patient safety will be impacted by this process. 

## Role of authors 

BGW is the inventor and constructor of the 10-D assessment concept and is responsible for the composition of this report. SB has given input to the report concerning specific clinical and scientific aspects relevant to the implementation of these peer review guidelines by peer reviewers, review boards, and ethics commissions and regarding the accuracy of the content in the report in general. 

## Conflict of interest 

Barry Woodcock is Editor-in-Chief of the *International Journal of Clinical Pharmacology and Therapeutics* published by Dustri-Verlag Dr. Karl Feistle GmbH & Co. KG, Munich-Deisenhofen, Germany, and Dustri-Verlag Inc., Rockledge, FL. USA. 

## Appendix 

### Registration and publication of observational studies in the International Journal of Clinical Pharmacology and Therapeutics 

Policy Statement: 2015/JF/BGW based on [9]. 

a) Current policy of the International Journal of Clinical Pharmacology and Therapeutics (IJCPT) permits the publication of registered and nonregistered observational studies.* 

b) Authors submitting observational studies for publication in IJCPT are asked to observe the recommendations made in the STROBE statement [10]. The STROBE recommendations are aimed at improving the clarity of study reporting. 

c) Authors are therefore required to describe in their papers exactly what they did during their studies and to explain the scientific background and rationale for the investigation being reported. 

Information should be provided on: 

i) Origins, motivations, and data interrogation methods used in the work (where applicable). 

ii) The study hypothesis: If the study hypotheses were developed after carrying out the investigation, authors will need to a) explain the steps taken to minimize bias, b) provide study protocols if they exist. 

*Under review (at June 2017)
